# Complete genome and comparative analysis of Xanthomonas oryzae pv. oryzae isolated from northern Thailand

**DOI:** 10.1099/acmi.0.000986.v4

**Published:** 2025-06-30

**Authors:** Atirada Boondech, Phatthira Ainmani, Anurak Khieokhajonkhet, Thanita Boonsrangsom, Pongsanat Pongcharoen, Tepsuda Rungrat, Kawee Sujipuli, Kumrop Ratanasut, Niran Aeksiri

**Affiliations:** 1 Center for Agricultural Biotechnology, Faculty of Agriculture, Natural Resources, and Environment, Naresuan University, Phitsanulok 65000, Thailand; 2 Department of Agricultural Sciences, Faculty of Agriculture, Natural Resources, and Environment, Naresuan University, Phitsanulok 65000, Thailand

**Keywords:** diversity, genome, Thailand, virulence, *Xanthomonas oryzae*, *Xanthomonas oryzae* pv. *oryzae* (Xoo)

## Abstract

Rice (*Oryza sativa L*.) is a vital global crop with a predominant presence in Asia, including Thailand. However, it faces a significant threat from bacterial blight disease, primarily caused by *Xanthomonas oryzae* pv*. oryzae* (*Xoo*). This research aims to provide valuable insights into the genetic virulence factors and genomic variations of *Xoo* strains isolated in Thailand. Furthermore, we present the first complete genomic database of Thai *Xoo*, offering a comprehensive resource for studying pathogen diversity, tracking virulence evolution and supporting disease management strategies in rice production. Our phylogenetic analysis unveils that the 20 Thai strains align with the Asian strains, setting them apart from African and US strains. Remarkably, the average nt identity values, in comparison with *Xanthomonas oryzae* type strain 35933 (XO35933), consistently exceed 99%. These strains can be classified into three assigned ribosomal sequence types. Our investigation into the pangenome and the phylogenetic relationships of these 20 *Xoo* genomes reveals a diverse genetic landscape, with the pangenome comprising 11,872 orthologous gene clusters, of which roughly 30% form the core genome. Notably, all of these genomes exhibit a clustered regularly interspaced short palindromic repeats-Cas I-C array, indicative of their adaptive immune mechanisms. All strains belonged to BXO1 type LPS cassette with high identity. Furthermore, our analysis identifies two distinct types of plasmids, namely, *Xanthomonas oryzae* pv. *oryzicola* strain GX01 plasmid pXOCgx01 (A46, A57, A83, A112, D and E) and the *X. oryzae* strain AH28 plasmid pAH28 (A97). This genomic resource will be valuable for advancing research on surveillance, prevention, management and comparative studies of this critical pathogen in the future.

## Data Summary

All whole-genome sequencing data have been uploaded to the National Center for Biotechnology Information (NCBI) public repository. The genome project is available under the BioProject ID PRJNA964490.

## Introduction

Bacterial blight (BB), primarily caused by *Xanthomonas oryzae* pv. *oryzae* (*Xoo*), is a devastating disease affecting significant economic losses on rice production globally [1]. *Xoo* is capable of infecting both primary rice subspecies, *Oryza sativa* subsp. *japonica* and *Oryza sativa* subsp. *indica*. However, the virulence of *Xoo* extends to a variety of host species, causing substantial economic losses due to bacterial disease, particularly in Asian regions. The organism has demonstrated its adaptability through the identification of over 30 different races of *Xoo *that were identified by researchers across various countries [2]. The primary approach to managing BB has been developing rice varieties resistant to *Xoo* through breeding programmes. Remarkably, researchers have characterized at least 42 BB disease resistance genes in rice, namely, Xa1, Xa3/Xa26, Xa4, Xa5, Xa10, Xa13, Xa21, Xa23, xa25, Xa27 and Xa41(t) [3]. The ability to infect plants relies on a combination of many virulence factors, including adhesins, polysaccharides and degradative enzymes. The type III secretion system is a main component in delivering these factors into plant cells [4].

Transcription activator-like effectors (TALEs) are the most abundant effectors in *Xanthomonas oryzae*, with up to 19 different TALEs in *Xoo* and 29 in Xoc strains [5,6]. TALEs are modular proteins comprised of three distinct domains, namely, the N-terminal domain, the C-terminal domain and the central repeat domain. The N-terminal domain contains the type III secretion signal. The C-terminal domain acts as nucleus localization and transcriptional activation. The central repeat domain is conserved. Within each repeat, it comprises 33–35 aa, in which there are two highly variable aa, typically located at positions 12 and 13 called the repeat variable di-residues (RVDs). While aa 13 is the primary contact point for the DNA base, aa 12 often plays a supporting role in stabilizing the RVD loop and contributing to the overall DNA binding affinity [7,9].

In Thailand, the investigation of *Xoo* diversity was carried out through the combined use of two methodologies including amplified fragment length polymorphism and repetitive sequence-based PCR. This study focused on northern Thailand, led to the classification of *Xoo* into six distinct lineages and categorized based on geographical locations [10]. Additionally, the genomes of two specific strains, SK2-3, isolated in Sukhothai Province, Thailand, and X-280, isolated in Andhra Pradesh, India, were compared. An intriguing discovery emerged, highlighting the striking similarity between these two genomes. This resemblance extended not only to the presence of TALE genes but also to the clonal lineage. Furthermore, a critical observation was made regarding the major S gene, SWEET11, and its interaction with the TALE genes of each strain. This interaction exhibited robust effectiveness, even in cases where it was hampered by the xa5 gene [11].

While substantial progress has been made in understanding *Xoo*, a critical knowledge gap persists regarding the comparative genomics of Thai strains in a global context. This study aims to fill this void by conducting a comprehensive genomic analysis of Thai *Xoo* strains. By elucidating the genetic diversity and characteristics of these strains, we seek to gain deeper insights into *Xoo*’s virulence and pathogenesis. The resulting genomic resource will serve as a valuable foundation for future research, including surveillance, prevention, management and comparative studies of this significant pathogen.

## Methods

### Genomic DNA preparation and sequencing

In this research, 20 *Xoo* isolates were collected from the lower northern region of Thailand. All were cultured by using a nutrient broth medium (peptone 10 g l^−1^, beef extract 3 g l^−1^, NaCl 5 g l^−1^ and pH 7.0) at 28 °C for 72 h. *Xoo* on nutrient agar forms yellow, circular, mucoid and shiny colonies with smooth margins within 24–48 h at 28–30 °C. First, DNA extraction was carried out using NucleoSpin Microbial DNA Mini kit from Machery and Nagel following the manufacturer’s guidelines. The DNA purity and concentration were assessed using a NanoDrop 2000c spectrophotometer and a Qubit from Thermo Fisher Scientific. Subsequently, whole-genome sequencing was conducted on an Illumina NovaSeq 6000 platform, which was conducted by Novogene Bioinformatics Technology Co., based in Beijing, China. The standard read configuration for bacterial whole-genome sequencing is paired-end 150 bp (PE150) with a sequencing depth of ≥100× for bacterial genomes. Meanwhile, Oxford Nanopore Technology was performed for long-read sequencing. The library was prepared from 400 ng per sample using the Ligation sequencing gDNA – Native Barcoding Kit 24 V14 (SQK-NBD114.24 kit, Oxford Nanopore Technologies, UK). The total library was loaded into R10.4.1 flow cells. All samples were performed on the MinION MK1B device and the MinKNOW software (v1.13.1) for up to 72 h. Reads with an average Phred quality score below Q10 were discarded. Additionally, to ensure sufficient read length for accurate downstream analysis, reads shorter than 1 kb were excluded as well.

### Whole-genome assembly, annotation and identification

The bioinformatic analysis was carried out using the public server usegalaxy.eu [12]. The quality of paired-end reads was assessed using FastQC v0.72 [13]. To enhance data quality, all reads underwent trimming and cropping using Trimmomatic v0.38.0 [14]. Adapter sequences were removed (ILLUMINACLIP:TruSeq3-PE:2 : 30 : 10), low-quality bases were trimmed (SLIDINGWINDOW:4 : 20) and reads shorter than 50 bp were discarded. Meanwhile, NanoPlot v1.44.1 was used to determine the quality of the raw reads [15] and Filtlong 0.2.1 to filter and trim long-read sequencing data with only reads longer than 1,000 bp and a quality score (Q) greater than 9 [16]. The hybrid genomic assembly was performed using Unicycle v0.5.0 [17] with the default setting. Pilon 1.24 was used to polish genome assemblies [18]. QUAST v5.2.0 [19] and BUSCO 5.5.0 [20] are two essential tools used together for evaluating genome assembly quality. For gene prediction and annotation, the genomes were annotated using the NCBI’s Prokaryotic Genome Annotation Pipeline (PGAP; https://www.ncbi.nlm.nih.gov/genome/annotation_prok/). The genomes were compared and aligned against the *X. oryzae* type strain 35933 (XO35933) by progressiveMauve alignment. This comparative analysis helps identify similarities and differences between the isolated strains and the reference strain. The entire genome project, along with the associated data and analysis, has been submitted to the National Center for Biotechnology Information (NCBI) public repository. The genome project is available under the BioProject ID PRJNA964490.

### Phylogenetic analysis, genome characterization and feature annotation

The study employed a variety of bioinformatic tools and methods for the comprehensive analysis of the *Xoo* genomes. CSIPhylogeny was used with *Xanthomonas* reference genomes to perform whole-genome SNP-based phylogenetic reconstruction [21]. This technique helps in understanding the genetic relatedness and evolutionary history of the *Xoo* strains. The CVTree3 web server (http://cvtree.online/v3/cvtree/) was employed to analyse the phylogenetic relationships among the *Xoo* strains [22]. This server uses a composition vector approach for phylogenetic analysis. The neighbour-joining (NJ) method was applied to construct phylogenetic trees based on genetic data. The K-tuple length used for this analysis was set to 6. The resulting phylogenetic trees were edited and visualized using FigTree v1.4.4 [23] and mega v11.0 [24]. For sequence typing and accurate strain grouping, ribosomal MLST (rMLST) and PubMLST species ID were employed [25]. These methods help in classifying strains based on sequence data. The average nuclotide identity (ANI) was calculated using JSpecies v3.9.6 [26]. ANI values provide insights into the genetic similarity between different strains, aiding in taxonomic classification. Roary v3.13.0 was used to perform pangenome analysis [27]. This analysis helps identify core and accessory genes among the *Xoo* strains, providing insights into shared and strain-specific genetic elements. The AntiSMASH 5.0 web server was utilized to classify potential secondary metabolite biosynthetic gene clusters (BGCs) [28]. These clusters are associated with the production of secondary metabolites, which can have various biological functions. The carbohydrate-active enzyme (CAZYme) annotation of protein sequences was conducted using dbCAN2 [29]. This analysis identifies enzymes involved in carbohydrate metabolism, including glycoside hydrolases (GHs), glycosyltransferases (GTs), carbohydrate-binding modules (CBMs), carbohydrate esterases (CEs) and polysaccharide lyases (PLs). The clustered regularly interspaced short palindromic repeats (CRISPR) arrays within the *Xoo* strains were characterized using the CRISPRCasTyper v1.6.2 server [30]. CRISPRs are involved in the bacterial immune system and can provide insights into the strain’s history of encounters with phages and plasmids.

TALE annotation using AnnoTALE involves identifying, analysing and classifying TALEs within sequenced genomes. The AnnoTALE suite (v1.5) provides a systematic pipeline for the detection, characterization and classification of TALEs.

These comprehensive bioinformatic analyses provide a deep understanding of the genetic diversity, virulence factors and functional elements within the *Xoo* strains, contributing to our knowledge of this pathogen and its potential impact on rice production.

### Plasmid analysis and antimicrobial-resistant genes

Plasmid sequence comparison was analysed by performing blastn searches against a complete plasmid database from the NCBI database. Antimicrobial resistance (AMR) gene identification was performed using ResFinder v4.1, utilizing the ResFinder and PointFinder databases for acquired resistance genes and chromosomal point mutations, respectively. The analysis was conducted using default parameters, including a minimum identity threshold of 90% and a minimum alignment coverage of 60%. Sequences with a minimum length of 60 bp were considered for resistance gene detection [31]. Additionally, fIDBAC was applied for supplementary characterization, including the identification of plasmid replicons and the contextual localization of resistance genes on chromosomal or plasmid regions. fIDBAC was used in the study for additional characterization or analysis related to plasmids or AMR genes [32].

## Result

### Genomic and phylogenetic profile

**Fig. 1.**
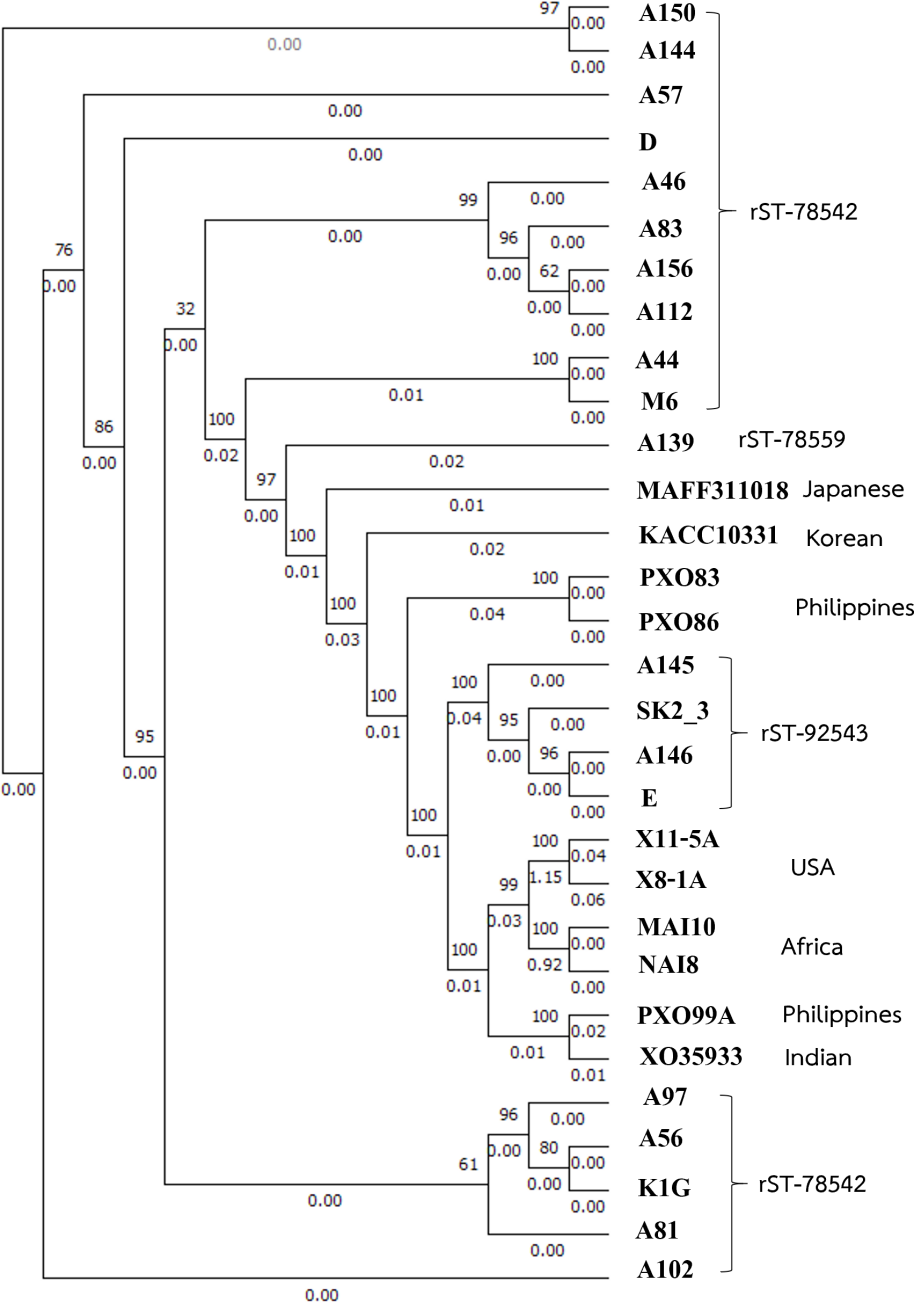
Phylogenetic analysis of *Xoo* strains and reference species based on whole-genome sequences, with corresponding rMLSTs in different colour. Genome sequences were analysed using the CVTree3 web server, and phylogenetic trees were constructed using the NJ method with a K-tuple length of 6 [22]. The tree was visualized using FigTree [23].

The complete genomes of 20 *Xoo* strains were successfully sequenced by Illumina and Nanopore sequencing as part of this study. These genomes were hybrid assembled from raw reads. Tables S2 and S3 (available in the online Supplementary Material) show the quality of sequences. Notably, these *Xoo* strains demonstrated uniformity in genome size and gene count. Moreover, the GC% content of all the assembled genomes fell within the typical range of 63–64% for *Xoo* isolates, as detailed in Table S1. The bacterial genome sizes showed in the range of 4.13 to 4.24 Mbp. The study also assessed the ANI values, revealing that these values exceeded 99% when compared with the *X. oryzae* type strain 35933 (XO35933). This ANI parameter surpasses the widely accepted 96% cut-off used to distinguish new species [33]. For the purpose of phylogenetic analysis, the researchers compared the genomes of these isolates with reference *Xanthomonas* species. The findings from the phylogenetic analysis revealed a close relationship between all the Thai *Xoo* strains and Asian strains (Fig. 1). These Asian strains included those from India (XO35933), the Philippines (PXO99A, PXO83 and PXO86), Japan (MAFF311018) and Korea (KACC10331). This Thai cluster was notably distinct from strains found in Africa (MAI10 and NAI8) and the USA (Xoc BLS256). To further enhance the understanding of the genetic relationships within this cluster, rMLST was applied, and the results were illustrated within brackets, providing additional insights into the genetic diversity of these Thai strains.

The rMLST analysis, which focused on 53 ribosomal *rps* and *rpl* genes (as detailed in Table S4), provided valuable insights into the genetic diversity among *Xoo* strains. These analyses resulted in the classification of these strains into three distinct groups, as summarized in Table S1. Specifically, a majority of the strains, including A44, A46, A56, A57, A81, A83, A97, A102, A112, A144, A150, A156, D, K1G and M6, were grouped together under the rST-78542 profile. Additionally, strains A145, A146, E and SK2_3 formed another group classified as rST-92543. Interestingly, one isolate, A139, exhibited a unique rST profile, designated as rST-78559.

**Fig. 2.**
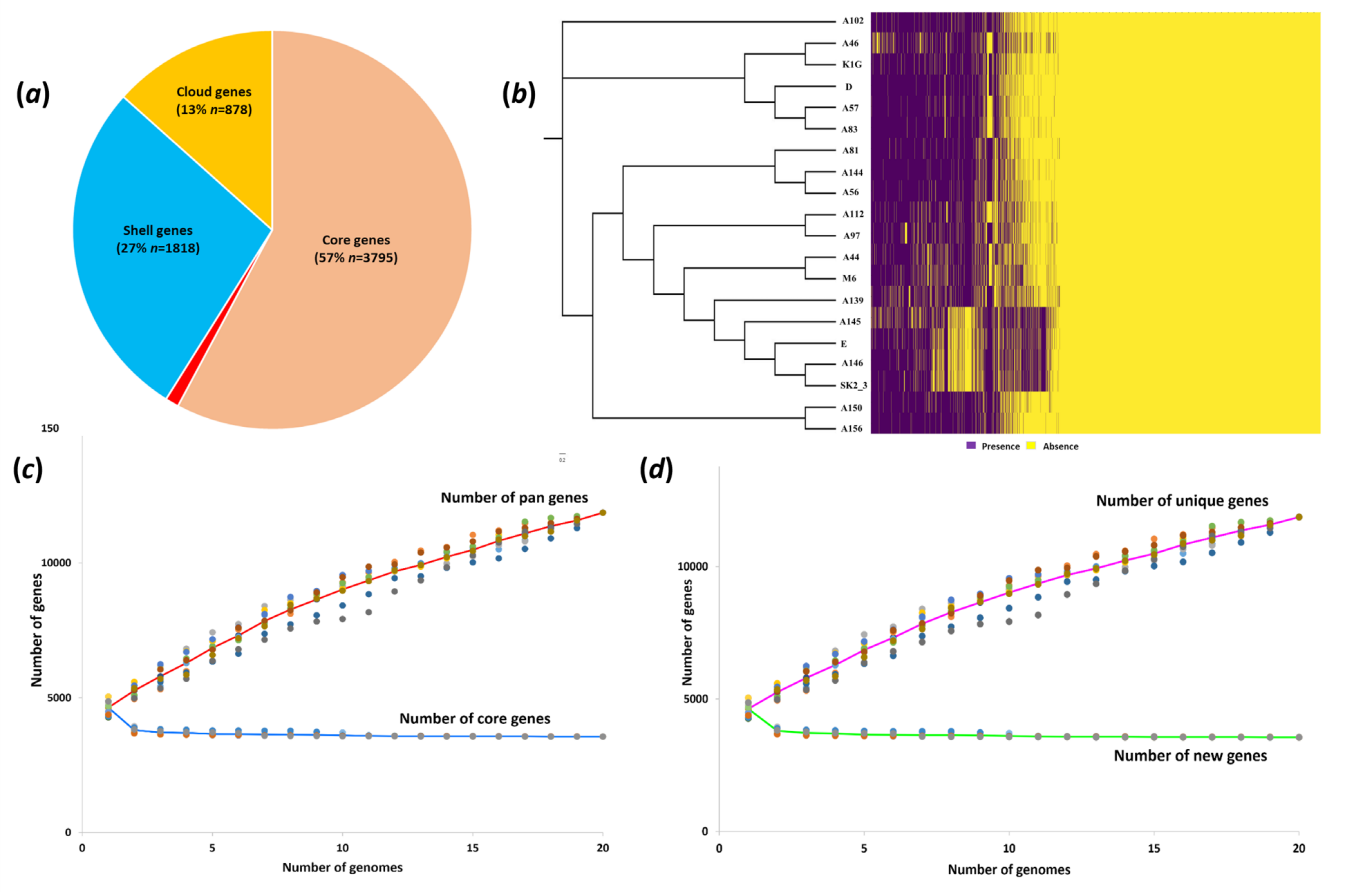
Visualization of pangenome analysis conducted with Roary for 20 isolates. (***a***) The composition of core, shell and cloud genes in the pangenome. (***b***) The presence/absence of all identified genes, with presence indicated in blue and absence in yellow. It also shows the clustering of samples based on gene presence/absence. (***c***) The core and pangenome development plots with the red line representing the number of pan-genes and the blue line representing the core genes. (***d***) The number of unique genes (purple line), which are genes exclusive to individual strains, and new genes (green line), i.e. genes not previously identified in compared genomes, as additional genomes are included.

We conducted a comprehensive pangenome analysis using Roary to delineate core, accessory, dispensable and unique genomes. The core genome comprises genes common to all *Xoo* strains (Fig. 2), comprised of 3,795 genes, while the soft-core genes numbered 72. Interestingly, only 58.92% of the entire species’ pangenome consisted of the combined core and soft-core genes, totalling 3,867 genes. The accessory genome included shell genes found in at least 15% but less than 95% of all strains (1,818 genes) and Cloud genes found in fewer than 15% of the strains (878 genes, accounting for 13.4% of the pangenome). Fig. 2b, a heatmap illustrating gene presence or absence across 20 genomes, enables the classification of strains into four distinct clusters. Additionally, Fig. 2c presents the core and pangenome development plots of *Xoo*, demonstrating that the pangenome is an ‘open’ system, with an increasing number of genomes leading to a growth in pangenome genes and a reduction in core-genome genes. Notably, Fig. 2d emphasizes the steady increase in novel and unique genes as more genomes were included.

**Fig. 3.**
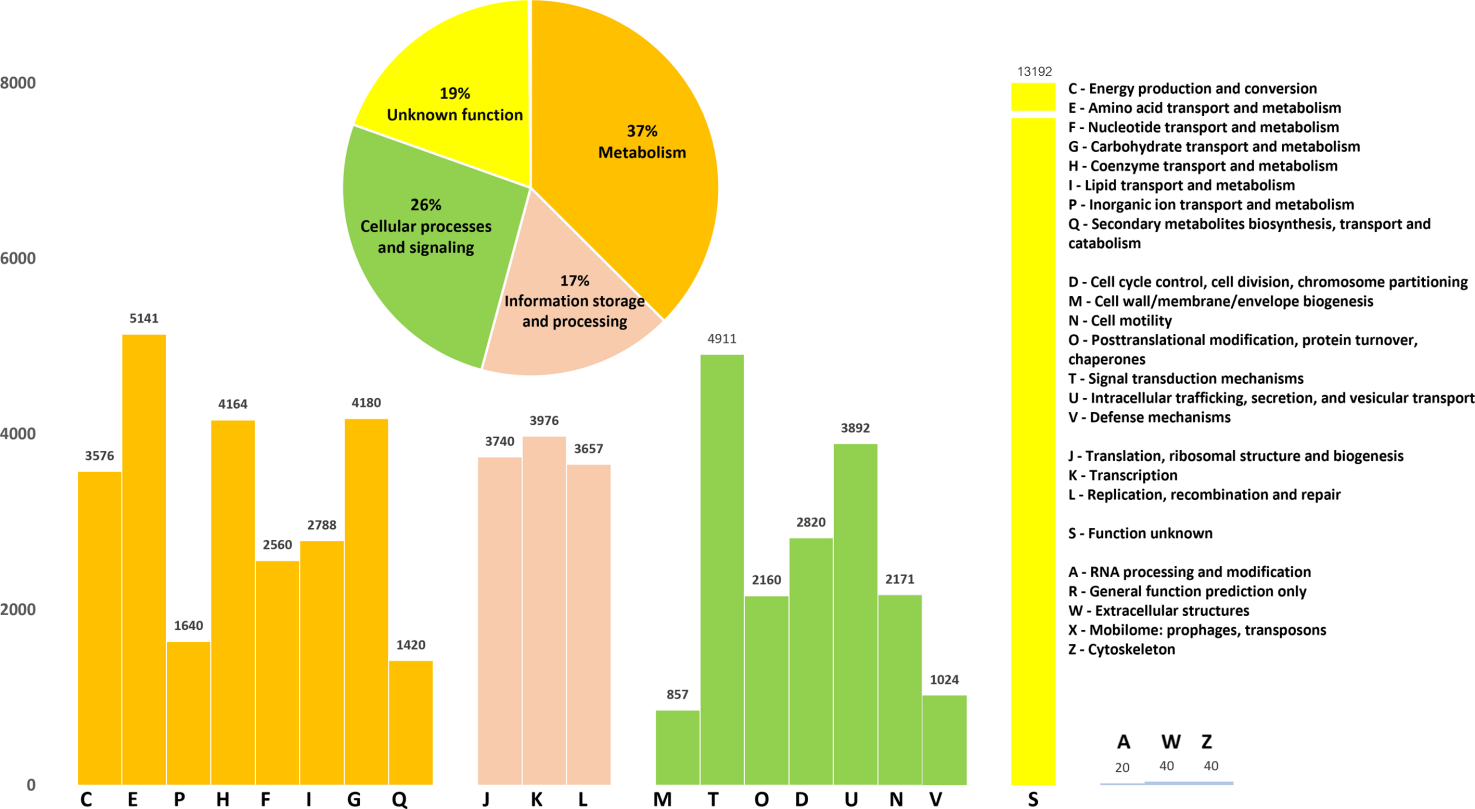
Functional categories of core genes according to COG classification. The annotation was performed using eggNOG and the COG databases to predict likely functions. Genes were categorized into 21 different COGs.

We utilized the Clusters of Orthologous Groups (COGs) of the protein database to classify functional genes (Fig. 3). The aim was to analyse the distribution of functional genes across the 20 *Xoo* strains, with a focus on those associated with bacterial virulence. Approximately 70% of the predicted coding sequences were assigned to 1 of the 21 COG categories, while about 19% of the genes remained functionally uncharacterized. Core genes were mainly linked to metabolism (37.47%), cellular processes and signalling (26.24%) and information storage and processing (16.73%). Approximately 19.4% of the core genes had unknown functions. Furthermore, we performed an analysis to identify potential secondary metabolite BGCs (Table S5).

### Comparison of the total number of CAZymes

**Fig. 4.**
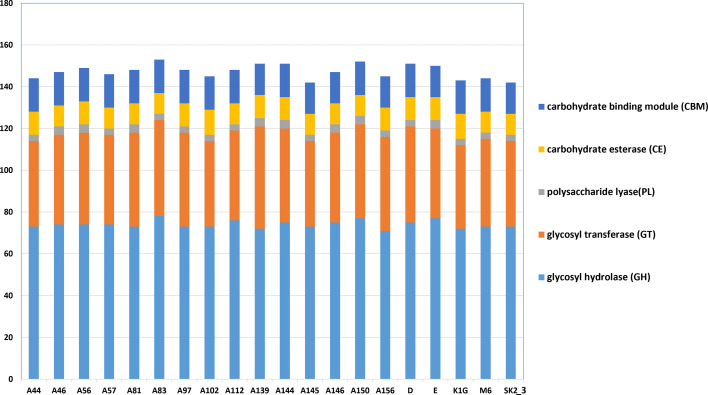
Comparative analysis of predicted CAZymes in genomes. CAZymes were categorized into five enzymatic domain classes: GH domain class, GT domain class, PL domain class, CE domain class and CBM domain class.

We conducted an analysis of the CAZyme repertoire in the *Xoo* strains, categorizing CAZymes into five major functional classes based on their aa sequence similarity (Fig. 4). The distribution of CAZymes across these functional classes was remarkably consistent among the strains. Among the characterized CAZymes, ~47–51% were GHs, 27–32% were GTs, 9–11% were CBMs, 6–8% were CEs and 1–2% were PLs. However, no auxiliary activity enzymes were identified in the genome, suggesting the absence of ligninolytic or oxidative carbohydrate degradation capabilities. Notably, this analysis revealed consistent proportions of all CAZyme groups across the *Xoo* strains.

### Variation of virulence genes

This study underscores the significance of conducting a phylogenetic analysis of pathogenicity determinants, fitness factors and virulence attributes to gain insights into strain variations. Plant cells possess a recognition mechanism for various microbial signature molecules, such as flagellin and LPSs, collectively referred to as Microbial- or Pathogen-Associated Molecular Patterns (MAMPs/PAMPs) [34]. Our analysis focused on studying variations in well-known genes associated with either PAMPs (such as flagellin and LPS cassettes), damage-associated molecular patterns (DAMPs, exemplified by the cellobiosidase gene) or the type III effectome, which interacts with PAMP-triggered immunity (PTI).

The *flic* gene, responsible for encoding flagellin and serving as a PAMP [35], exhibited no variation in our analysis, displaying 100% identity among all strains. Furthermore, we examined the presence of the *raxX* gene, which encodes a peptide linked to the resistance gene *Xa21* [36]. Mutations in the *raxX* gene have been associated with disease development in *Xa21*, compromising the host plant resistance. However, it is noteworthy that we detected the *raxX* gene with no mutation.

**Fig. 5.**
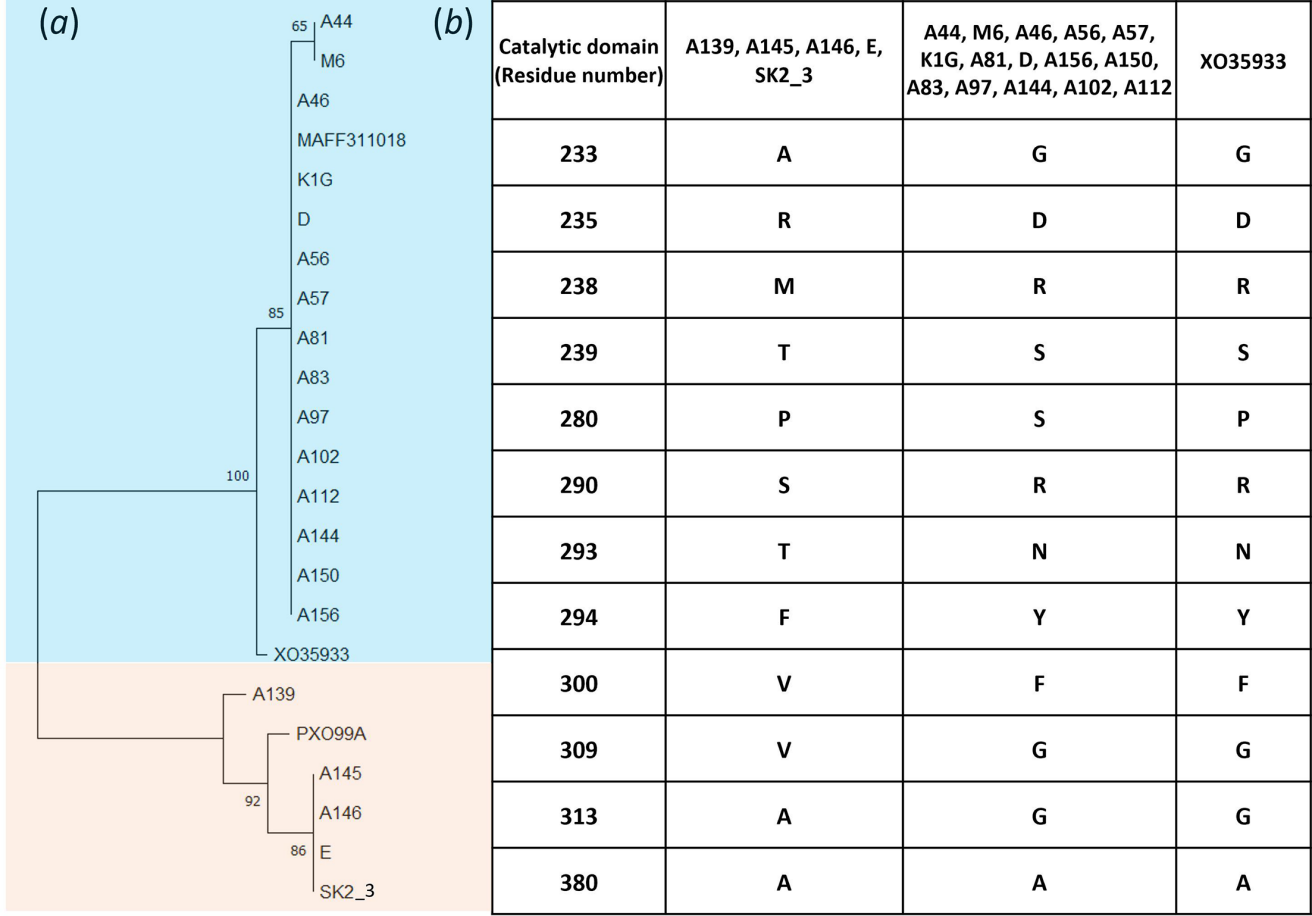
Phylogenetic assessment of the cellobiosidase-encoding gene. Protein sequences of cellobiosidase were aligned, and a phylogenetic tree was constructed using the NJ method. Bootstrap values at the nodes denote the percentage from 500 replicates. The scale bar (0.002) represents the number of aa substitutions per site. Details of variations in the aa residues of the catalytic domain are presented in tabular format.

Cellobiosidase is a crucial pathogenicity determinant secreted via the type II secretion system, known for inducing programmed cell death and initiating the innate immune response in rice tissue [37]. Our analysis focused on the *cbsA* gene, represented in the phylogenetic tree (Fig. 5). Notably, the *cbsA* gene can be categorized into 2 distinct alleles, and we outlined the positions of 12 aa residues that are either similar or different between these alleles (Fig. 5).

**Fig. 6.**
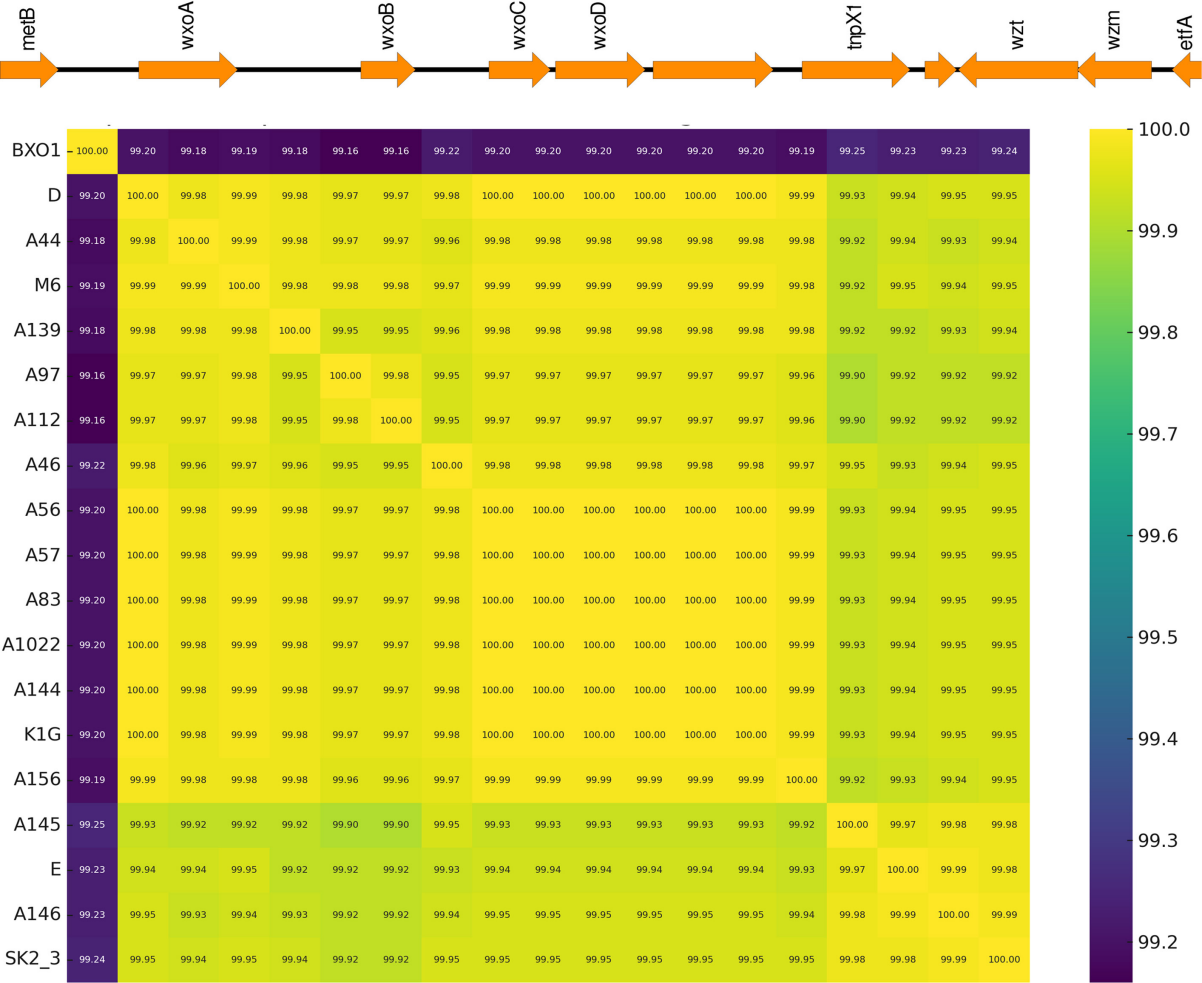
Comparative heatmap of gene similarity across strains. The heatmap depicts the percentage similarity of LPS genes across various strains. The colour scale ranges from purple (lower similarity, ~99.2%) to yellow (higher similarity, ~100%).

The variation in LPS gene clusters among bacterial strains, as observed in *Xoo*, highlights the genetic diversity and adaptability of the organism. Two types of LPS cassettes have been identified in *Xoo*, namely, BXO1 and BXO8. Notably, all strains analysed were classified as BXO1-type LPS cassettes, which displayed highly conserved regions across most strains, with nearly 100% similarity, as illustrated in Fig. 6. This heatmap effectively highlights the genetic similarity and conservation levels among strains for these genes.

**Fig. 7.**
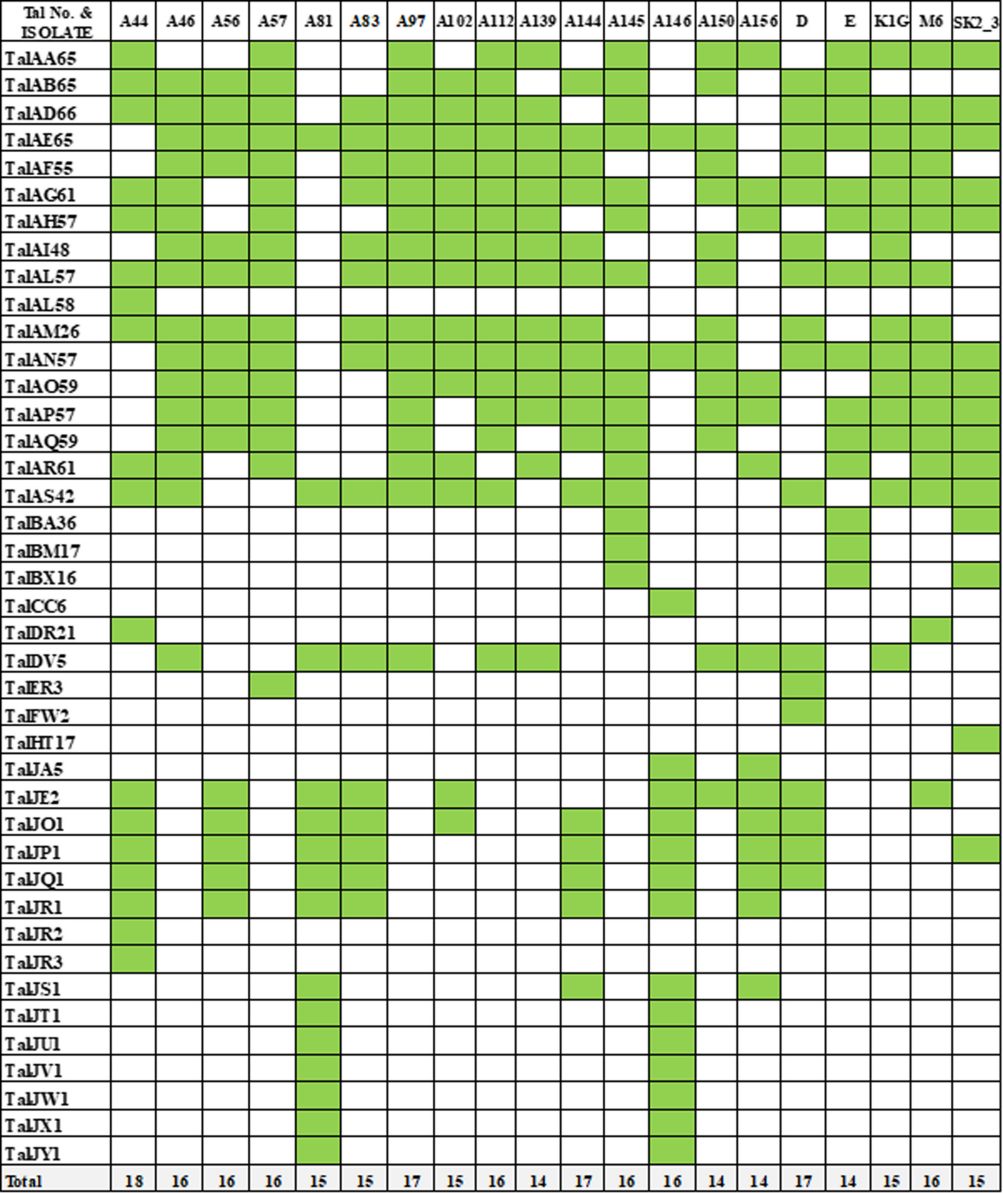
Distribution of TALE genes across strains. This matrix displays the presence (green) or absence (white) of TALE genes across different strains.

In bacterial disease pathology and virulence, type III effectors (T3Es) play a significant role [38]. T3Es are represented in Supplemental Table S6, showing that AvrXa10 and pthXo1 show variability, indicating that their activity may depend on specific conditions or stimuli. Meanwhile, OmpR and sctC were low across all strains, with values of 1–2, indicating consistent detection but low abundance. These TALEs often exhibit a repetitive nature, making identification challenging. We identify TALE by using AnnoTALE as shown in Fig. 7. There were 18 for a higher diversity of TALES, whereas TALEs on average had 13. Strains like A44 and A81 show a higher diversity of TALE genes, potentially reflecting broader genetic variation or adaptability, while A56 and A57 primarily share their genes with others, indicating a lesser degree of uniqueness. Many TALE genes, like TalAH57, TalAE65 and TalJE2, are found across multiple strains, indicating they might play a broader role across strains. Some strains have unique genes, such as TalAL58 and TalJO2 (in strain A44) and TalKJ1 and TalKK1 (in strain A83), which could be strain-specific adaptations or serve specialized functions. Moreover, we successfully classified the 24 non-TALEs of *Xoo*, using the categorization available on https://euroxanth.ipn.pt/. Additionally, we identified the T1-6SS and extracellular enzymes (Table S6), with the noteworthy finding that there were no significant differences among them.

### CRISPR-Cas systems

CRISPR represents a bacterial adaptive immune system [39,40]. In all *Xoo* strains examined, we found the presence of CRISPR type I, which includes key components like cas3, cas5, cas8c, cas7, cas4, cas1 and cas2. The direct repeats associated with these CRISPR sequences are consistently 31 bp in length. Our results revealed variations in the number of spacer sequences, ranging from 65 to 94 across the strains. Detailed CRISPR sequences and spacer counts are provided in Table S7.

### Plasmid detection and antimicrobial analysis

The analysis of raw reads included a search for the presence of plasmids, which was conducted using plasmidSpades v3.15.3 [41]. This examination revealed plasmid sequences in seven strains within the dataset. Contigs exceeding a size of 5 kb were subsequently subjected to manual blastn analysis against the complete plasmid database. The findings uncovered two distinct types of plasmids among these strains. The identified plasmid was *Xanthomonas oryzae* pv. *oryzicola* strain GX01 plasmid pXOCgx01 [42], which was present in strains A46, A57, A83, A112, D and E. *X. oryzae* strain AH28 plasmid pAH28 [43] is found in strain A97. Detailed information regarding the lengths of these plasmids and their corresponding blast summaries can be found in Table S8. Additionally, previous research had indicated that *Xoo* exhibited resistance to certain bactericides commonly used, including streptomycin, bismerthiazol and amobam. In our analysis of AMR genes, we identified the presence of the *soxR*, *nalD*, *MexT* and *adeL* genes, which are associated with antibiotic efflux resistance mechanisms. All genes were classified within the resistance-nodulation-division of efflux pump systems. Further details can be found in Table S9.

## Discussion

This study sought to comprehensively characterize *Xoo* isolates obtained from rice in Thailand, focusing on their pathogenicity, virulence, fitness and genetic factors. To achieve this, a range of analytical tools was applied to assess the genomic makeup of these isolates. The population analysis of these genomes revealed that the Thai *Xoo* strains were closely related to Asian strains, clearly distinguishing them from isolates found in the USA and Africa. This phylogenetic insight into pathogenicity and virulence gene variation is instrumental for further research. The study investigated a range of genes linked to MAMPs/PAMPs (such as *raxX* and *flic*), DAMPs (including the cellobiosidase gene) and the T3E, which interacts with PTI. Repeats (CRISPR) represents a bacterial adaptive immune system. Its mechanism involves incorporating and storing genetic sequences from invading threats like bacteriophages and harmful plasmids as spacers. Over time, the CRISPR cassette can evolve, providing immunity against phages through the utilization of LPSs as receptors. Additionally, it acts as a barrier to horizontal gene transfer mediated by extrachromosomal elements like plasmids. Therefore, assessing the variability of CRISPR in *Xoo* strains holds significant importance. This comparative genome analysis of Thai *Xoo* strains contributes significantly to our understanding of *Xoo* pathogenicity and its coexisting species. These genomic differences could contribute to variations in host specificity and disease severity. The findings offer valuable insights for future investigations into genome virulence and pathogenesis. More importantly, the findings from this study provide a foundational dataset for establishing a complete genomic database of Thai *Xoo*. Furthermore, this research expands our knowledge of effective strategies for managing BB disease, particularly through the development of resistant rice varieties in breeding programmes.

## Supplementary material

10.1099/acmi.0.000986.v4 Supplementary Material 1.

## Abbreviations

AMR: antimicrobial resistance
ANI: average nt identity
BB: bacterial blight
BGCs: biosynthetic gene clusters
CAZYme: carbohydrate-active enzyme
CBM: carbohydrate-binding module
CE: carbohydrate esterase
COGs: Clusters of Orthologous Groups
CRISPR: clustered regularly interspaced short palindromic repeats
DAMPs: damage-associated molecular patterns
GH: glycoside hydrolase
GT: glycosyltransferase
LPS: lipopolysaccharide
MAMP: microbial-associated molecular pattern
NJ: neighbour joining
PAMP: pathogen-associated molecular pattern
PL: polysaccharide lyase
PTI: PAMP-triggered immunity
rMLST: ribosomal MLST
RVD: repeat variable di-residue
TALE: transcription activator-like effector
T3E: type III effector
Xoo: Xanthomonas oryzae pv. oryzae
